# Performance Level Affects Full Body Kinematics and Spatiotemporal Parameters in Trail Running—A Field Study

**DOI:** 10.3390/sports11100188

**Published:** 2023-09-28

**Authors:** Matteo Genitrini, Julian Fritz, Thomas Stöggl, Hermann Schwameder

**Affiliations:** 1 Department of Sport and Exercise Science, University of Salzburg, 5400 Hallein-Rif, Austria; 2 Adidas AG, 91074 Herzogenaurach, Germany; 3 Red Bull Athlete Performance Center, 5303 Thalgau, Austria

**Keywords:** trail running, biomechanics, incline running, ecological study

## Abstract

Trail running is an emerging discipline with few studies performed in ecological conditions. The aim of this work was to investigate if and how biomechanics differ between more proficient (MP) and less proficient (LP) trail runners. Twenty participants (10 F) were recruited for a 9.1 km trail running time trial wearing inertial sensors. The MP athletes group was composed of the fastest five men and the fastest five women. Group differences in spatiotemporal parameters and leg stiffness were tested with the Mann–Whitney U-test. Group differences in joint angles were tested with statistic parametric mapping. The finish time was 51.1 ± 6.3 min for the MP athletes and 60.0 ± 5.5 min for the LP athletes (*p* < 0.05). Uphill sections: The MP athletes expressed a tendency to higher speed that was not significant (*p* > 0.05), achieved by combining higher step frequency and higher step length. They showed a tendency to shorter contact time, lower duty factor and longer flight time that was not significant (*p* > 0.05) as well as significantly lower knee flexion during the stance phase (*p* < 0.05). Downhill sections: The MP athletes achieved significantly higher speed (*p* < 0.05) through higher step length only. They showed significantly higher knee and hip flexion during the swing phase as well as higher trunk rotation and shoulder flexion during the stance phase (*p* < 0.05). No differences were found with respect to leg stiffness in the uphill or downhill sections (*p* > 0.05). In the uphill sections, the results suggest lower energy absorption and more favorable net mechanical work at the knee joint for the MP athletes. In the downhill sections, the results suggest that the more efficient motion of the swing leg in the MP athletes could increase momentum in the forward direction and full body center of mass’ velocity at toe off, thus optimizing the propulsion phase.

## 1. Introduction

Trail running is a strongly emerging endurance running discipline [1]. It has been previously defined as any running that takes place in an open country on unpaved surfaces (i.e., off road) with <20–25% paved surface [2]. Mountains represent the ideal scenario with mild, steep or technical uphill (UH) and downhill (DH) traits, where athletes must cope with both physical and mental fatigue. Trail running competitions cover many distances from five up to several hundred kilometers. In the last two decades, the number of studies focusing on the biomechanics and physiology of graded running (i.e., running on non-level conditions, on negative/positive inclines) grew in parallel with race attendance [3,4,5,6]. Several lab-based studies reported differences in spatiotemporal parameters, kinematics, kinetics and muscle activation when comparing level and graded running [7]. Nonetheless, a major shortcoming of lab settings is the absence of interaction between sport performers and the external environment, which is critical to any outdoor discipline. In fact, in a lab-based setting, several factors critical for trail running may be hardly reproduced, such as the irregularity of the ground and different terrains (gravel, grass, etc.), to name a few. Therefore, field-based investigations may convey valuable information to coaches and practitioners. Novel wearable technology has enabled the research of trail running outside the laboratory. For example, Defer et al. [8] used inertial sensors to report that footwear with a smaller heel-to-toe drop may elicit changes in the foot-strike pattern and improve performance in short DH runs; Giandolini et al. [9] used portable EMG sensors to investigate neuromuscular fatigue during ecological DH running, reporting both central and peripheral fatigue-induced dysfunctions. Björklund et al. [10] utilized pressure insoles to investigate the kinetics in a trail running field test, reporting a higher force impulse in UH sections and higher peak force in DH sections. Born et al. [11] reported trail running intensity to be accurately reflected by the tissue saturation index, measured with a compact near-infrared spectroscopy device during a trial run in outdoor conditions. Always in an ecological framework, Townshend et al. [12] found running speed to depend more on stride length than on stride frequency. However, in comparison to other endurance disciplines, little is known about if and how biomechanics differ between trail runners of different performance levels. To the best of our knowledge, only one study investigated such differences in running performance for level and UH running on a treadmill [13]. The authors reported more proficient (MP) trail runners to express lower (better) cost of running, lower step frequency, lower leg stiffness and lower vertical stiffness compared to less proficient counterparts during level running at the same speed. In the same study, no differences were observed in uphill running biomechanics, whereas there still exists no evidence about possible different biomechanical behavior of trail running athletes of different performance levels in downhill running. During competitions, trail runners of different performance levels were reported to differ in both absolute and relative speed (as a fraction of average race speed) with more proficient athletes running UH sections at lower relative speed and DH sections at higher relative speed compared to less proficient counterparts [14]. Therefore, it is possible that movement pattern and related mechanical parameters differ between trail runners of different performance levels when running at self-selected speed in ecological conditions. New insights in this regard combined with previous findings may represent an important step towards the development of specific training protocols aiming to (I) prevent injuries and (II) optimize performance in a growing endurance discipline. Therefore, the purpose of this study was to investigate if and how spatiotemporal parameters, full-body kinematics and leg stiffness differ between performance levels in a field trail running session. It was hypothesized that MP athletes would express different kinematics and spatiotemporal parameters (STP) as well as lower leg stiffness compared to less proficient (LP) counterparts.

## 2. Materials and Methods

### 2.1. Participants and Experimental Design

Twenty subjects (10 M, 10 F) were recruited from local trail running associations (age [years]: 32.8 ± 8.3 M, 33.4 ± 8.1 F; body height [cm]: 177.2 ± 6.0 M, 166.3 ± 6.9 F; body mass [kg]: 71.9 ± 5.8 M, 61.6 ± 6.9 F; experience in trail running [years]: 3.3 ± 1.5 M, 4.1 ± 1.2 F). This study was approved by the ethical committee of the local university (GZ-10/2022). The inclusion criteria required that participants

- were between 18 and 50 years old,
- had at least 1 year experience in trail running,
- had a minimum training frequency of 2 times per week,
- ran a minimum weekly training volume of 30 km,
- had sustained no injuries in the previous 3 months prior to the study.

**Figure 1:**
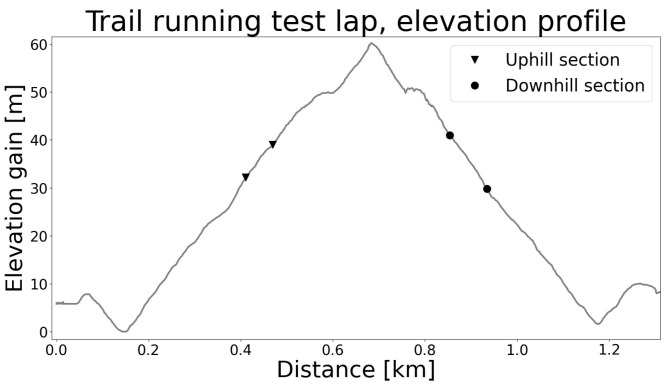
Elevation profile of trail running test lap; a full test consisted of 7 repetitions. Each lap presents one UH and one DH section, where data were retained for further analysis.

After providing their informed consent, anthropometric data, including body height, body mass and length of relevant body segments, were recorded. On a separate day, the participants completed a 9.1 km trail running time trial consisting of 7 laps of the same 1.3 km route (Figure 1). Each lap presented an ascent of 60 m, resulting in a 420 m gain across the entire trial. Before the test, the participants were accompanied during a complete lap of the running route to familiarize themselves with the test environment followed by a warm-up at a self-selected intensity, consisting of short UH and DH runs and static stretching exercises. During the test, time and positional data, STP, full-body kinematics and leg stiffness were recorded. The performance level was determined according to the finish time: the fastest 5 men and the fastest 5 women were assigned to the more proficient MP athletes group; the slowest 5 men and the slowest 5 women were assigned to the LP athletes group.

### 2.2. Materials

During the trail running test, the participants wore a GPS watch (Garmin Forerunner 935), a foot sensor to record STP (Stryd Summit Powermeter; Stryd, Inc., Boulder, CO, USA, sampling frequency 1 Hz) and a full-body motion-capture system (Xsens Link, Xsens Technologies BV, Enschede, The Netherlands). The latter consisted of 15 inertial measurement units (IMUs, model MTx, size 36 × 24.5 × 10 mm, mass 10 g, sampling frequency 240 Hz); IMU sensors were located on the head, shoulders (2×), arms (2×), forearms (2×), thighs (2×), legs (2×), feet (2×), sternum and pelvis.

### 2.3. Data Analysis

The data from specific UH and DH sections were retained for further analysis. Those sections (Figure 1, slope ca 12%, length = 80 m UH and 120 m DH) were selected so as not to present abrupt changes in steepness and ground morphology. Step frequency (SF), step length (SL), contact time (CT) and flight time (FT) were measured with the Stryd foot sensor. STP were made adimensional in order to control for anthropometric differences between the participants [15]. In particular, SF was scaled with $\sqrt{g/l_{0}}$; SL was scaled with *l*_0_; and CT and FT were scaled with $\sqrt{l_{0}/g}$, where *l*_0_ is the body height of each subject and *g* is the gravity acceleration. Duty factor (DF) is the percentage amount of time spent in CT during a stride, where stride indicates the time between two consecutive ipsilateral foot strikes. DF was calculated from the adimensional CT and FT as DF = 100 × CT/(2(CT + FT)). It was assumed that gait phases have similar duration for both legs with contralateral foot strike occurring at 50% of gait cycle. The joint angles of the ankle, knee, hip, trunk and shoulder were processed with the software MVN Analyze^©^ (Xsens Link, Xsens Technologies BV, Enschede, The Netherlands) in HD mode and No-Level scenario, thus yielding joint angle trajectories as recommended for biomechanical applications [16] and validated in previous works [17]. In particular, the No-Level scenario is well suited when investigating joint angles specifically rather than other quantities (e.g., center of mass trajectory). Also, in HD mode, the software processes data over a larger time window to obtain an optimal (and more consistent) estimate of the position and orientation of each body segment, ultimately resulting in a precise estimation of joint angle trajectories [16]. Leg stiffness was measured with the Stryd foot sensor. Validation studies reported that such a portable device provides values comparable to those obtained with gold-standard methods [18]. All subsequent analyses were performed in a Python environment. Gait events were identified with a previously validated algorithm [19]. Gait cycle data were resampled to 100 data points. For the laps 2–6 (the first and last were discarded, thus resulting in 5 retained laps) in each UH and DH section, the first 20 strides per leg for each subject were analyzed.

### 2.4. Statistics

Separately for the UH and DH sections and for each participant, the data from all strides were averaged. The graphical representations for STP and leg stiffness are provided as boxplots, while joint angles are represented as time series against gait cycle percentage. Group differences in STP and leg stiffness were assessed via the Mann–Whitney U-test, alpha = 0.05. Group differences in joint angle kinematics were assessed with statistical parametric mapping [20], *t*-test with alpha = 0.05.

## 3. Results

One female participant was not able to complete the trail running test, thus resulting in a sample size of ten for MP athletes (5 M, 5 F) and nine for LP athletes (5 M, 4 F).

The general characteristics of the MP and LP athletes are presented in Table 1. Body mass was significantly higher for the LP athletes (*p* < 0.05), while height, age and experience in trail running did not differ (*p* > 0.05).

The finish times for the trail running test as well as the split times for the specific UH and DH sections where the present data were collected are presented in Table 2. The finish time for the trail running test and split times in the DH sections were significantly lower for the MP athletes (*p* < 0.05). In the UH sections, the difference between the groups was not significant (*p* > 0.05).

### 3.1. Spatiotemporal Parameters

**Figure 2:**
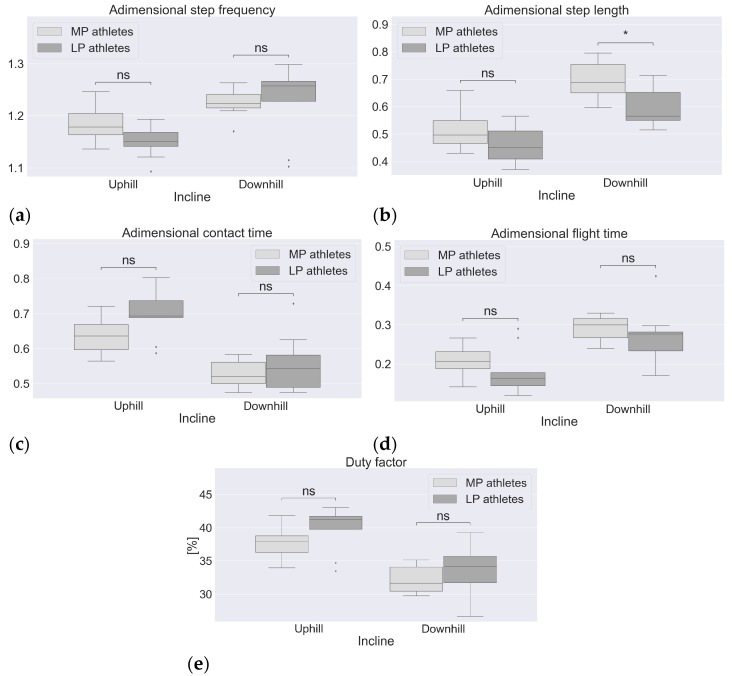
Spatiotemporal parameters by terrain and performance level. MP = more proficient athletes, LP = less proficient athletes, ns = no significant difference between groups, * = *p* < 0.05. Subfigures: (**a**) step frequency; (**b**) step length; (**c**) contact time; (**d**) flight time; (**e**) duty factor.

**Figure 3:**
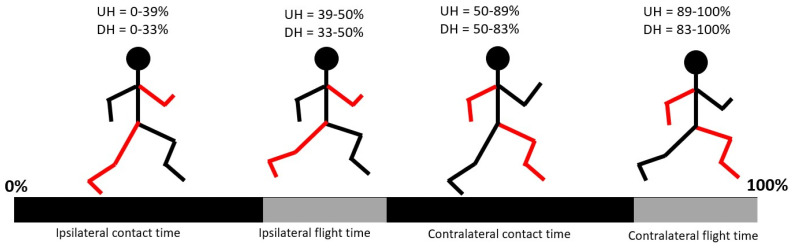
Duration of gait phases with respect to gait cycle in uphill (UH) and downhill (DH) conditions. Values are averaged across all participants to provide a general overview, as differences in DF between the MP and LP athletes were not significant and the values differ by only ca 2–3% between performance levels.

No significant differences (*p* > 0.05) were found with respect to SF in the UH or in the DH sections (Figure 2a). The MP athletes showed significantly longer SL in the DH sections only (*p* < 0.05, Figure 2b). No significant differences were found in CT and FT (*p* > 0.05) despite that the data distribution of the MP athletes showed generally lower values for CT in the UH sections and higher values for FT in both the UH and DH sections (Figure 2c,d). No significant differences (*p* > 0.05) were found with respect to DF in the UH or DH sections despite that the data distribution of the MP athletes showed generally lower values, especially in the UH sections (Figure 2e). The overall average DF was 39% in the UH sections and 33% in the DH sections. A schematic representation of the gait phases during a complete gait cycle is provided in Figure 3.

### 3.2. Kinematics

Significant differences were found at the shoulder, trunk, hip and knee in the DH sections in both the stance and swing phases (*p* < 0.05). Also, significant differences were found at the knee joint in the UH sections in the early stance and late swing phases (*p* < 0.05). No differences were found for the ankle joint (*p* > 0.05).

**Figure 4:**
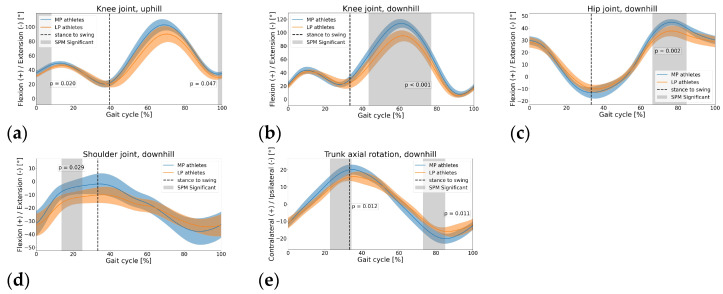
Kinematic comparison between performance levels. (**a**–**c**): lower body; (**d**,**e**): upper body. MP = more proficient athletes, LP = less proficient athletes, SPM = statistical parametric mapping.

***Uphill:*** The MP athletes expressed significantly higher knee flexion (*p* < 0.05) during the initial stance phase and very late swing phase (Figure 4a). ***Downhill*** *Hip and knee:* Significantly higher peak flexion (*p* < 0.05) was observed during the swing phase for the MP athletes (Figure 4b,c). *Shoulder:* The MP athletes showed significantly higher flexion (*p* < 0.05) during the mid-late stance phase compared to the LP athletes (Figure 4d). *Trunk:* Significantly larger ROM (due to larger peak values) was expressed by the MP athletes (*p* < 0.05) compared to that of the LP athletes (Figure 4e).

### 3.3. Leg Stiffness

**Figure 5:**
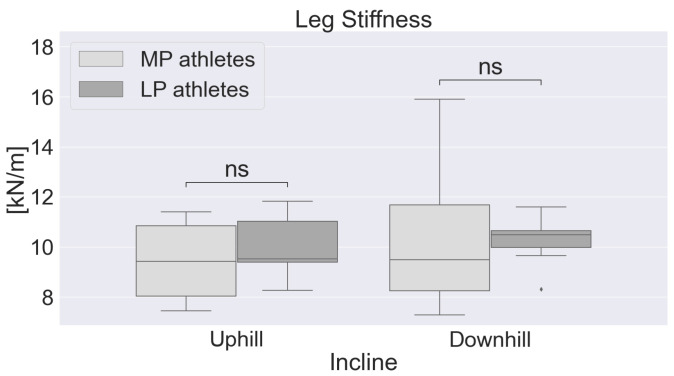
Leg stiffness separated by performance level in UH and DH sections. MP = more proficient athletes, LP = less proficient athletes, ns = not significant difference between groups.

No significant differences (*p* > 0.05) for leg stiffness were found between the athletes of different performance levels, neither in the UH sections nor in the DH sections (Figure 5).

## 4. Discussion

The present results confirmed the hypothesis that full-body kinematics would differ between athletes of different performance levels. The hypothesis that STP would differ between athletes of different performance levels was confirmed in DH only. Finally, the hypothesis that leg stiffness would be lower for MP athletes was not supported by our results. In the present study, body mass was significantly lower in the MP athletes. This is in line with a previous investigation that compared trail runners of different performance levels in a laboratory setting with elite athletes showing lower body mass compared to the LP counterparts [13]. The present results show how kinematics and STP differ between performance levels in outdoor trail running. To the best of our knowledge, this is the first study to report on full-body kinematics across a whole trail running field test.

The MP athletes presented a systematic tendency to longer SL with a significant difference in the DH sections, while no significant differences were found for SF between performance levels (Figure 2a,b). Townshend et al. [12] found that running speed was mainly regulated by SL rather than by SF; this appears to apply to a greater extent to the MP athletes. In fact, the MP athletes showed less SF variation between the UH and DH sections and more SL variation between the UH and DH sections compared to the LP athletes. In both the UH and DH sections, SL appears strongly related to flight time (Figure 2b–d present the same trend). This is consistent with previous works that focused on level running and reported that the significantly longer flight time for MP athletes was associated with a tendency to longer step length [21]. The relation between shorter CT, longer SL and higher running speed of the MP athletes appears to apply to the UH sections only, consistent with previous works [22].

Previous investigations that compared MP and LP athletes with a treadmill UH running test, tested participants of both groups at the same running speed(s) [13,22,23] rather than at a self-selected pace as in the current study; therefore, caution is needed when comparing the results. Besson et al. [13] observed neither significant differences nor clear tendencies for SF, SL, FT and CT. Instead, Padulo et al. [22] reported significantly longer FT and shorter CT at all tested speeds for MP athletes; similarly, Garcia et al. [23] reported a non-significant yet systematic trend of MP athletes to longer FT while running at different UH steepnesses at the same speed. Our results are consistent with these studies in that the MP athletes running at self-selected speeds exhibited a clear tendency to shorter CT in the UH sections and longer FT in both the UH and DH terrains. We observed that the not significant but yet clear tendency to higher running speed of the MP athletes in the UH sections (Table 2) results from the combination of higher SF and longer SL. This is most likely achieved with a more efficient propulsive phase during shorter CT and lower DF. Shorter CT, lower DF and better propulsion of the MP athletes are possibly related to the higher knee-joint flexion at foot strike and during the early stance phase (Figure 4a); during the stance phase, such difference implies a significantly lower knee-joint ROM during the flexion sub-phase (~0–15% gait cycle) and a similar ROM during the extension sub-phase (~15–39% gait cycle) compared to the LP athletes. We suggest that the lower ROM to be covered during the flexion sub-phase elicits shorter CT and positively influences propulsion. Previous laboratory-based investigations [24] reported a systematic trend with higher running speeds in UH being associated to larger knee ROM during stance and higher energy absorption (in particular, during the flexion sub-phase, i.e., eccentric phase) and lower energy generation (during the extension sub-phase, i.e., concentric phase). Given this kinematic–kinetic link, our results suggest that there could be lower energy absorption for MP athletes (due to lower ROM during the flexion sub-phase) and similar energy generation (due to similar ROM during the knee-joint extension sub-phase), resulting in more favorable net mechanical work and less demand upon knee extensors in the eccentric phase (i.e., flexion sub-phase) during the stance phase. Such factors would ultimately contribute to a higher running speed and better running economy. Padulo et al. [25] highlighted the importance of CT in terms of the athlete–environment interaction in determining mechanical and metabolic performance. This was also observed in a recent outdoor trail running study where MP athletes, in both the UH and DH sections, showed higher coordination variability (CV) in both the upper and lower body during the stance phase than the LP counterparts [26]. The authors suggested that the lower CV observed in the LP athletes is most likely the consequence of a higher muscular pre-activation used to stiffen some joints in order to cope with the surface irregularity. This may, in turn, result in a less efficient propulsive phase during a longer CT, thus negatively affecting FT, SL and, ultimately, running speed.

In the DH sections, the MP athletes achieved significantly higher running speeds with significantly longer SL despite a slightly lower SF (Figure 2a,b). The higher SL is the result of a more efficient propulsive phase for the MP athletes with a similar CT between the groups (Figure 2c). With the distribution of the gait phases of Figure 3 in mind, the MP athletes express higher hip and knee joint flexion with the swing leg during the contralateral stance phase. Previous studies showed that, as running speed increases, the peak flexion angles at the hip and knee joint of the swing leg increase as well [27]. Furthermore, it was reported that a higher peak flexion at the hip joint of the swing leg in elite sprinters is associated with a higher forward speed of the full-body center of mass compared to sub-elite counterparts [28]. Combining these results, we suggest that the higher hip and knee joint flexion during the swing phase may result in a more forward displacement of the center of mass of the swing leg in the sagittal plane. In turn, this would contribute to an increase in the forward momentum of the full-body center of mass, thus increasing speed in the forward direction during the contralateral stance phase. Ultimately, this would result in a higher speed at toe off. Finally, a higher forward speed during a not significantly different FT (Figure 2c) would ultimately result in a longer SL, thus explaining the higher speed achieved by the MP athletes. Other factors possibly contributing to a more efficient propulsive phase in the MP athletes are a higher self-confidence due to systematic DH running training or superior motor adaptability while running on irregular terrains [26]. Earlier studies [29] that focused on level running reported that, at a higher running speed during the swing phase, higher knee extensor torque is needed to bring the leg forward as well as higher hip flexor torque to reverse the hip extension occurring in the late stance phase (because of higher angular momentum elicited by higher angular velocity). However, such considerations do not seem to apply to overground DH running since the larger hip and knee joint ROM shown by the MP athletes during the swing phase occur during a longer time compared to the LP athletes (tendency to longer FT and lower SF for the MP athletes). This suggests that the MP athletes may not express higher angular velocity at the hip and knee joints and, consequently, hip flexor and knee extensor torques are similar between the groups. Future studies may address this specific aspect. In contrast to the swing phase, no differences during the stance phase were found at the hip and knee joints between the MP and LP athletes. Previous investigations [24] found that, while running at 10% DH, increased speed was associated with increased hip ROM and increased energy generation, energy absorption, peak moment and peak power. With this kinematic–kinetic relationship, the absence of significant differences at the hip suggests that MP and LP athletes do not adopt different strategies for hip energy and power generation/absorption. As for the knee joint, a previous study [24] did not find a clear relation between kinematics and kinetics; therefore, based on the kinematic data here, it is not possible to speculate on possible group differences in knee joint kinetics during the stance phase. In the DH sections, significant differences were found in upper-body kinematics during both the stance and swing phases. During the mid and late stance phase, the shoulder corresponding to the stance leg was significantly more flexed, and the torso was significantly more contralaterally rotated for the MP athletes (i.e., rotated in the direction of the swing leg, Figure 4d,e). The higher shoulder flexion is most likely a strategy to accentuate trunk axial rotation. Higher trunk contralateral axial rotation during the stance phase is needed in order to maintain null the full-body angular momentum about the vertical (longitudinal) axis. In fact, as previously mentioned, greater flexion at the hip and knee joints of the swing leg would displace the center of mass of the swing leg of the MP athletes further forward during the contralateral stance. Nonetheless, such movement would also elicit a higher axial rotation in the ipsilateral direction for the lower body (i.e., in the direction of the stance leg), compensated by higher contralateral rotation of the upper body. Similarly, previous works that focused on level running [30] reported an increase in upper limb angular momentum with an increase in running speed to compensate for increased lower limb angular momentum during the swing phase.

Lower leg stiffness has been associated with more experienced athletes [13,31]. However, in this study, no differences were found between the different performance levels, and similar values were found in the UH and DH sections (Figure 5). This is not surprising for the DH sections as no differences during the stance phase were found in the joint angles of the lower limbs. As for the UH sections, the lower ROM of knee joint flexion during the stance phase for the MP athletes would suggest higher stiffness for this group; nonetheless, no differences were observed between the performance levels.

Trail running is a multifaceted sport, and many factors other than biomechanical have been reported to impact performance. Trail running performance is determined with factors such as VO_2max_ [10,32] and body composition [10]. Other authors highlighted the importance of maximal aerobic speed in determining performance in mountain ultramarathons [33]. To better understand the relationship between biomechanics and physiology in a field study, future investigations may integrate these two aspects by equipping participants with both a full-body motion-capture system and a portable metabolic cart. Nonetheless, a trade-off between comprehensiveness of research design and obtrusiveness of equipment should be pursued, as field performance may otherwise be impaired.

This study presents a few limitations: first, the presence of both male and female athletes in the two performance level groups may make our findings less sex-specific, as male and female athletes were reported to differ in specific parameters such as CT, SF and leg stiffness [13]. Nonetheless, we suggest that the homogeneous distribution of males and females in the two groups of the MP and LP athletes may counterbalance such shortcomings, thus making the comparison between performance levels appropriate. Second, the self-selected speed during the test has the disadvantage of making the results more difficult to reproduce. Nonetheless, it also has the advantage of being the most realistic conditions when simulating a race, thus better highlighting group differences.

## 5. Conclusions

The present field study showed that the performance level differentiates the full-body kinematics and spatiotemporal parameters in trail running. In the uphill sections, the more proficient runners showed a tendency for higher speed, resulting from a combination of higher step frequency and step length. Their kinematics suggest lower energy absorption and more favorable net mechanical work at the knee joint compared with the less proficient runners. In the downhill sections, the more proficient athletes achieved a significantly higher speed, resulting from only higher step length. Their kinematics suggest a more efficient motion of the swing leg, which would contribute to increased momentum in the forward direction of the full-body center of mass, thus optimizing the propulsion phase. There was no effect of the trail running performance level on leg stiffness, either in the uphill or the downhill sections. Future studies may combine motion-capture and EMG measurement in the field to further clarify the relationship between kinematics and specific muscle activity/fatigue. Such findings may be of relevance for coaches and practitioners to integrate training protocols for specific muscle groups when optimizing running kinematics. This would help to lower the injury risks in trail running.

## Figures and Tables

**Table 1:** General characteristic of MP and LP athletes. * = *p* < 0.05, ns = not significant.

	MP Athletes	LP Athletes	Significance
Height [cm]	170.7 ± 9.1	173.6 ± 7.7	ns
Body mass [kg]	63.3± 6.7	71.1 ± 7.9	*
Age [years]	32.9 ± 8.0	33.3 ± 8.4	ns
Experience [years]	4.0 ± 1.4	3.3 ± 1.3	ns

**Table 2:** Time comparison between performance levels for the trail running test as well as for the specific sections of the trail running test where the present data were collected. * = *p* < 0.05, ** = *p* < 0.01, ns = not significant.

	MP Athletes	LP Athletes	Significance
Finish time [min]	51.1 ± 6.3	60.0 ± 5.5	*
DH sections [s]	36.0 ± 5.0	42.0 ± 3.3	**
UH sections [s]	29.8 ± 4.4	33.3 ± 3.8	ns

## Data Availability

Data are available upon request to the authors.
